# Beyond Diagnosis: Aerosol Viral Monitoring to Transform Epidemic Responses

**DOI:** 10.34133/research.1059

**Published:** 2026-01-07

**Authors:** Peng Zhou, Yingming Xu, Amber N. McElroy, Mark J. Osborn, Al Dababneh, Raj Palraj, Despina Moschou, Charareh Pourzand, David A. Ellis, Dingguo Zhang, Ian H. White, Tianhong Cui

**Affiliations:** ^1^ University of Minnesota, Minneapolis, MN, USA.; ^2^ Mayo Clinic, Rochester, MN, USA.; ^3^ University of Bath, Claverton Down, Bath, UK.

## Abstract

Aerosol viral transmission monitoring enables early, noninvasive detection of infectious disease spread by identifying airborne viral particles in shared environments. Integrating sampling, sensing, and data analysis, this perspective outlines an integrated framework for transforming epidemic responses from reactive testing to proactive surveillance. It synthesizes current technological advances and deployment experiences to identify key challenges in detection efficiency, data management, and societal trust. This marks a shift from clinical diagnosis to environmental surveillance, transforming epidemic responses from testing individuals to monitoring shared air. By enabling earlier interventions, these systems help reduce transmission, protect vulnerable groups, and limit disruptions to daily life and the economy.

Data saves lives, and better data can save more lives. As emphasized by the U.S. Data Modernization Initiative (DMI), public health relies on timely and complete data, which are often difficult to obtain [1,2]. The COVID-19 pandemic exposed this limitation: Information derived from symptomatic patients is delayed, incomplete, and fails to capture the dynamics of spread. By the time cases are recorded, transmission may have already occurred [3]. Actionable intelligence, which enables effective intervention, requires moving data collection upstream to capture signals at the earliest stages of likely infection and spread. Aerosol-based viral transmission monitoring offers a promising possibility: Rather than asking whether individuals are sick, it detects whether pathogens, and potentially transmission, are present/taking place in shared environments. Through sampling, sensing, and real-time analysis, such systems could enable continuous shifting of epidemic response from reactive testing to proactive early warning [4]. When embedded into infrastructure, they could strengthen preparedness, but beyond the underpinning technology, their success depends as much on trust, governance, and integration with existing health systems as on technology. This perspective synthesizes current advances and real-world deployments of aerosol viral monitoring through literature review and case-based analysis to explore its potential role in future epidemic responses.

Aerosols, defined as fine liquid or solid particles suspended in air, are typically less than 5 μm in diameter and can remain aloft for prolonged periods with wide dispersement. Everyday human activities such as breathing, speaking, and coughing continuously generate these particles, and they are an efficient vehicle for respiratory pathogen spread [5]. Notably, a growing body of evidence indicates that multiple viruses, including influenza and severe acute respiratory syndrome coronavirus 2 (SARS-CoV-2), can spread via aerosols, a route whose role has long been underestimated and may even dominate under certain conditions. Infectious SARS-CoV-2 has, for instance, been directly recovered from aerosols in both clinical settings and animal models, with viable viruses detected even prior to symptom onset [6]. Testing individuals one by one with nasal or oral swabs in epidemic screening or public space management is neither practical nor sustainable. Therefore, aerosol monitoring offers a complementary approach. Rather than targeting individuals, it monitors the process of transmission itself: A positive signal means that infectious agents are present and potentially spreading in the environment. Detection would enable earlier and broader public health interventions.

Airborne transmission monitoring systems are typically composed of sampling, sensing, and data processing and management components. Sampling devices capture and concentrate rare biological particles from complex air environments, providing the basis for downstream detection. Sensor modules then detect and translate pathogen-associated signals into measurable outputs that can be interpreted. Data processing and management, in turn, extend beyond the instrument itself, enabling real-time analysis, communication among distributed nodes, and the delivery of results to public health authorities. Together, these components transform environmental trace into actionable information, establishing the scientific and technological foundation for pathogen transmission surveillance in air.

Sampling is the critical entry point of airborne transmission monitoring, tasked with capturing scarce viral particles from complex air environments and converting them into analyzable samples. This step is inherently challenging: Respiratory aerosols are highly polydisperse, fluctuate across time and space, and often occur at concentrations lower than background indoor particles. To address these constraints, a variety of devices have been developed. Inertial separation samplers (impactors, impingers, and cyclones) use momentum to divert particles from the airstream onto a collection medium, enabling high flow rates and aerodynamic size fractionation but often compromising sample quality during extended sampling. Filter samplers trap aerosols on porous membranes with high efficiency across size ranges, although recovery requires elution steps that reduce yield. Condensation growth collectors enlarge submicrometer particles through humidification and capture them in liquid, improving fine-particle efficiency and preserving infectivity, albeit with added complexity and environmental sensitivity. No single method performs optimally under all conditions, underscoring the long-standing trade-off between capture efficiency and biological integrity in various sampler designs [7]. Ongoing innovations are beginning to address this limitation, suggesting that future devices may overcome this trade-off and provide more reliable material foundations for downstream sensing and analysis.

Sensing is a critical step in aerosol-based monitoring, converting captured biological material into definitive signals. Conceptually, it can be described along 2 dimensions: recognition strategies and transduction mechanisms. Recognition strategies include nucleic acid assays such as polymerase chain reaction (PCR), isothermal amplification, or DNA binding platforms with high specificity (e.g., CRISPR); affinity capture of viral proteins or intact virions through antibodies, aptamers, or receptor mimetics; and particle-level physicochemical signatures derived from size, charge, or spectral fingerprints, with viability-aware variants bridging presence and infectiousness [8–11]. Distinguishing viable from nonviable viruses is essential, as only infectious particles indicate ongoing transmission, whereas signals from residual or inactivated fragments may persist after outbreaks and cause false alarms if misinterpreted. These strategies are coupled to diverse information transduction mechanisms, including electrochemical (current, impedance, and potential), optical (fluorescence, luminescence, and plasmonics), mechanical (cantilevers and quartz crystal microbalance/surface acoustic wave technology), or digital readouts such as barcoded sequencing [12,13]. State-of-the-art devices integrate recognition and transduction within microfluidic platforms, support multiplex detection, and incorporate quality controls for unattended operation [14]. Yet, multiple demands remain including achieving high sensitivity together with rapid time-to-result, maintaining specificity while ensuring robustness against inhibitors, and above all delivering low detection limits per unit time in fluctuating environments. A practical sensor is thus a layered system, co-designed across recognition strategies, transduction mechanisms, and data handling, to transform sparse aerosols into actionable epidemiological information.

Data are the core of transforming local detection signals into system-level public health information. In aerosol-based viral transmission monitoring, this role is fulfilled by data processing and management, which convert raw sensor signals into actionable epidemiological insight. The process begins with real-time signal analysis to remove noise, extract features, and generate accurate results. Integration typically occurs at 2 levels: within a single site, such as an airport hall, or data from multiple subsystems are aggregated to evaluate local conditions; across sites, signals from schools, hospitals, and transportation hubs that are further merged into a unified data stream [15,16]. Equally important is the transmission of results to public health authorities, where they can support early warning, epidemic modeling, and resource allocation. The associated challenges to realize the promise of such a network are considerable: processing large data flows with low latency, ensuring security and privacy, and maintaining interpretability so that results remain understandable and trustworthy even when algorithms are uncertain. Emerging approaches combine wireless connectivity, cloud platforms, and machine learning to identify patterns and forecast risks. Statistical process control frameworks, such as risk-adjusted control charts in healthcare systems, may also offer complementary approaches for continuous trend monitoring and anomaly detection [17]. Ultimately, effective data management transforms fragmented environmental signals into a coherent surveillance network. Establishing aerosol viral transmission monitoring within the broader architecture would transform public health preparedness. To illustrate how these technical components may be combined into practical systems, Fig. 1 provides an overview of an airborne disease monitoring framework. It highlights how sampling, sensing, and data analysis can be integrated into settings such as schools, airports, and shopping centers that have high and transient visitor frequencies, with results ultimately feeding into centralized public health decision-making infrastructure.

**Fig. 1.**
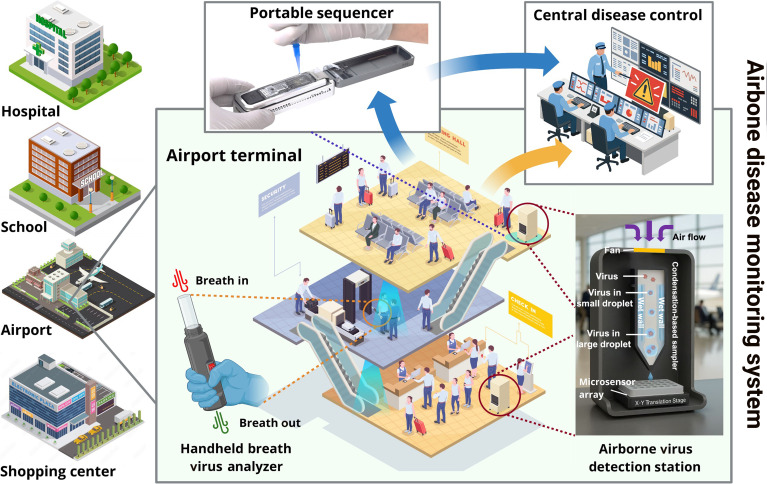
Overview of an airborne disease monitoring system. The framework integrates multiple components, including a floor-standing aerosol virus analyzer, portable breath analyzers, and sequencing, with data reporting to central disease control. Potential deployment scenarios include schools, airports, and shopping centers, demonstrating how laboratory technologies can be embedded into everyday public health infrastructure.

However, realizing this integrated system in practice requires overcoming deeper engineering and operational challenges. Pathogen aerosols are typically present at extremely low concentrations, making efficient sampling and sensitive detection difficult. Devices must maintain long-term stability and accuracy in diverse environments while also remaining feasible to operate, calibrate, and maintain in the field.

Yet, even if these hurdles are overcome, a final challenge remains transforming promising devices into durable infrastructure. A single sensor may capture a signal, but only when units are networked, standardized, and maintained do they begin to function as part of public health systems. The COVID-19 pandemic offered a lesson: The U.S. “Test to Treat” initiative showed that diagnostic tools gained value not from accuracy alone but from integration into services. For aerosol monitoring, the same principle holds across venues including airports or hospitals where multiple nodes must be linked to reflect the state of a broader environment. Across sites, shared protocols will need to also allow for ongoing calibration, maintenance, and quality control to ensure reliability. Infrastructure will have to evolve again when monitoring is embedded into schools and workplaces as it becomes an integral part of epidemic preparedness.

The promise of aerosol pathogen transmission monitoring not only lies in technical performance but also relies on whether it can be adopted, trusted, and sustained by society as a whole [18]. Public health innovations often falter when they neglect social determinants of health or exclude the voices of affected communities. Technologies developed in isolation risk widening inequities: Those most vulnerable are often the last to benefit, while unresolved concerns over privacy, accountability, and data use can erode trust. Building resilient monitoring systems therefore requires dialogue across 3 groups: researchers and healthcare providers who generate evidence and refine methods, policymakers who set governance and allocate resources, and the public who ultimately live with these systems in schools, workplaces, and transport hubs [19]. Patient and Public Involvement and Engagement (PPIE) provides a structured path for such dialogue, ensuring that questions of intrusiveness, data use, and barriers to adoption are addressed early and with community involvement. Embedding aerosol monitoring into infrastructure thus demands more than reliable sensors; it requires ongoing collaboration across science, policymakers, and society, supported by governance frameworks that safeguard privacy, ensure fairness, and foster transparency. Practical implementation should include robust data encryption, controlled data access, and clear consent protocols to protect individual and community privacy. Equally, co-designing deployment with local stakeholders and maintaining open communication channels can enhance trust, encourage participation, and ensure that monitoring serves public rather than institutional interests. Seen this way, PPIE reframes monitoring as a civic partnership rather than a technical imposition, aligning innovation with legitimacy.

Aerosol viral transmission monitoring transforms epidemic readiness: Rather than testing whether individuals are sick, it detects whether transmission is occurring in shared environments. This shift moves data collection upstream, offering a complementary layer to clinical diagnostics and enabling earlier, broader interventions. Its promise lies in turning the presence of infection into a collective signal of risk actionable at the level of communities and public spaces. To realize this vision, technical advances must be matched by governance, trust, and sustained collaboration. Future research will need to explore how these systems can be scaled, standardized, and integrated across diverse environments while ensuring data security and public acceptance.

Finally, this vision is far from theoretical and is beginning to find support in real-world deployments. Recent studies have shown that aerosol-based monitoring systems can be successfully operated in everyday environments such as schools, where they provided early signals of viral circulation that aligned with broader community infection trends [20]. These examples demonstrate that what was once confined to laboratory research is now moving toward integration into daily life, underscoring the potential of aerosol monitoring to become a durable and dynamic layer of public health infrastructure.
